# Diagnostic Performance of Biomarkers Urinary KIM-1 and YKL-40 for Early Diabetic Nephropathy, in Patients with Type 2 Diabetes: A Systematic Review and Meta-Analysis

**DOI:** 10.3390/diagnostics10110909

**Published:** 2020-11-07

**Authors:** Georgia V. Kapoula, Panagiota I. Kontou, Pantelis G. Bagos

**Affiliations:** 1 Department of Biochemistry, General Hospital of Lamia, End of Papasiopoulou, 35100 Lamia, Greece; gkapoula@hotmail.com; 2 Department of Computer Science and Biomedical Informatics, School of Science, University of Thessaly, Papasiopoulou 2-4, 35100 Lamia, Greece; pkontou@dib.uth.gr; 3 Department of Mathematics and Engineering Sciences, Informatics LAB, Hellenic Military Academy, 16673 Athens, Greece

**Keywords:** kidney injury molecule 1 (KIM-1), chitinase-3-like protein 1 (YKL-40), diabetic nephropathy, meta-analysis

## Abstract

There is a lack of prediction markers for early diabetic nephropathy (DN) in patients with type 2 diabetes mellitus (T2DM). The aim of this systematic review and meta-analysis was to evaluate the performance of two promising biomarkers, urinary kidney injury molecule 1 (uKIM-1) and Chitinase-3-like protein 1 (YKL-40) in the diagnosis of early diabetic nephropathy in type 2 diabetic patients. A comprehensive search was performed on PubMed by two reviewers until May 2020. For each study, a 2 × 2 contingency table was formulated. Sensitivity, specificity, and other estimates of accuracy were calculated using the bivariate random effects model. The hierarchical summary receiver operating characteristic curve hsROC) was used to pool data and evaluate the area under curve (AUC). The sources of heterogeneity were explored by sensitivity analysis. Publication bias was assessed using Deek’s test. The meta-analysis enrolled 14 studies involving 598 healthy individuals, 765 T2DM patients with normoalbuminuria, 549 T2DM patients with microalbuminuria, and 551 T2DM patients with macroalbuminuria, in total for both biomarkers. The AUC of uKIM-1 and YKL-40 for T2DM patients with normoalbuminuria, was 0.85 (95%CI; 0.82–0.88) and 0.91 (95%CI; 0.88–0.93), respectively. The results of this meta-analysis suggest that both uKIM-1 and YKL-40 can be considered as valuable biomarkers for the early detection of DN in T2DM patients with the latter showing slightly better performance than the former.

## 1. Introduction

Chronic kidney disease (CKD) in patients with diabetes mellitus (DM), also known as diabetic nephropathy (DN), is one of the most common microvascular complications of the kidneys [1,2], as well as the leading cause of end-stage kidney disease (ESKD) and is associated with an increased risk of death in general, mainly due to cardiovascular diseases [3].

There are multiple mechanisms involved in the development and progression of DN. Currently, persistent microalbuminuria, along with estimated glomerular filtration rate (eGFR), is widely used as a non-invasive screening for the disease. However, its diagnostic accuracy is limited by the fact that in the absence of glomerular proteinuria, it has been shown that tubular dysfunction can even proceed glomerular injury and thus, microalbuminuria [4]. Recently, inflammation has also been emerged as a key pathophysiological mechanism [5]. Therefore, in order to diagnose this pathological entity, there is an imperative need to identify effective, sensitive, and specific biomarkers that can predict the early development of DN. Several glomerular, tubular, and inflammatory markers have been recently identified as potential markers for DN such as neutrophil gelatinase-associated lipocalin (NGAL), N-acetyl-β-D-glucosaminidase (NAG), liver-fatty acid-binding protein (LFABP), urinary kidney injury molecule 1 (KIM-1), and chitinase-3-like protein 1 (YKL-40), but until now, none of them is currently established well enough to replace the gold standard biomarker, the urinary albumin/creatinine ratio (uACR). In a previous meta-analysis, we examined NGAL [6], a small protein (25KDa), measured in both serum and urine, as a potential biomarker for the diagnosis of the disease. Our results showed that it can be considered as a valuable biomarker for the early detection of DN in patients with diabetes type 1 and type 2.

KIM-1 is a type 1 epithelial transmembrane glycoprotein that is expressed after ischemia or toxicity from the proximal tubules of the kidney [7]. Recently, previous meta-analysis has suggested that urinary KIM-1 is a promising biomarker for early detection of acute kidney injury (AKI) [8]. Moreover, it has been reported that type 2 diabetic patients with normo- or microalbuminuria had mildly increased KIM-1 values that progressively increased during follow up [9].

In addition, YKL-40 is a 40 KDa chitin binding glycoprotein whose full biological functions are still unknown. It is considered as an inflammatory marker and indicator of endothelial dysfunction, which is found to be elevated in type 2 diabetes [10,11] and type 1 diabetes and is increased along with albuminuria [12].

On the basis of these findings, we performed a systematic review and meta-analysis in order to fully understand the diagnostic performance of uKIM-1 and serum/plasma YKL-40 for predicting early diabetic nephropathy in patients with type 2 diabetes.

## 2. Materials and Methods

### 2.1. Study Search Strategy

We systematically searched the literature in accordance with the preferred reporting items for systematic reviews and meta-analyses (PRISMA) [13]. Two independent reviews conducted the search in the Medline database up to end of May 2020 using the following combination of terms and keywords: (“KIM-1” OR “kidney injury molecule 1”) AND (“YKL-40” OR “chitinase 3-like protein 1”) AND (“Diabetic nephropathy” OR “diabetic kidney disease” OR “albuminuria”). In order to complement our electronic search, we further searched in other electronic engines such as Google Scholar. We also performed a manual search on the reference list of included papers and related reviews and checked for special congress abstracts in order to retrieve data from studies not identified through the search and may bias the meta-analysis result if not included [14]. Finally, in order to avoid local literature bias, no language restriction was applied [15].

### 2.2. Study Selection and Inclusion—Exclusion Criteria

All the included studies were screened for further selection. Two independent reviewers read the full text of the retrieved articles and separately extracted the data in a data collection form. The final selection of the target articles was again reviewed by a third investigator. In cases of multiple publications on the same research, those with the most complete and detailed data were included in the meta-analysis. Any disagreement was resolved by consensus between the three reviewers.

All articles included in the meta-analysis were required to meet the following inclusion criteria: (1) must have measurements of uKIM-1 and serum/plasma YKL-40 in healthy individuals and in patients with type 2 diabetes (T2DM) and normomicroalbuminuria; (2) the degree of DN must be determined using the following clinical index: urinary albumin/creatinine ratio (uACR) or urinary albumin excretion (UAE), according to the American Diabetes Association [16], categorizing the T2DM patients with normoalbuminuria when uACR < 30 mg/g; and (3) determination of uACR must be estimated using either 24 h urine sample or random spot urine sample (preferably the morning void).

### 2.3. Data Extraction

The data extracted for each study included: first author’s last name, year of publication, study location, gender, age of participants, eGFR (estimated glomerular filtration rate), glycated hemoglobin (HbA1c), uACR or UAE for the controls, and the T2DM patients with different stages of DN. In addition, the different kits used for the determination of uKIM-1 and serum/plasma YKL-40 were also recorded. Data concerning the quality of the sample collection processing and storage were additionally extracted. Measurements of urine KIM-1 and serum or plasma YKL-40 were recorded. Urine concentrations for KIM-1 were also recorded as normalized to the urinary creatinine concentration (uΚΙΜ-1/Cr). In order to construct the 2 × 2 contingency table, we also obtained true positive (TP), false positive (FP), true negative (TN), and false negative (FN) if provided, or equivalent data such as cut off values, sensitivity, specificity, positive predictive value (PPV), negative predictive value (NPV), and the area under the receiving operating curve (AUC). When multiple cutoffs were reported in a study with different specificity and sensitivity values, the data with the highest Youden index were included in the meta-analysis, as described in the statistics.

### 2.4. Assessment of Methodological Quality

The quality assessment of the studies was assessed using the Quality Assessment of Diagnostic Accuracy Studies 2 (QUADAS-2) tool [17], analyzed by the Review Manager Software (RevMan 5.2.3, The Cochrane Collaboration, 2020, UK). The QUADAS tool is a quality assessment tool specifically developed for systematic reviews of diagnostic accuracy studies and consists of four key domains: patient selection, index test, reference standard, and flow and timing, and each domain is rated as low risk, high risk, and unclear risk.

### 2.5. Statistical Analysis

We conducted data synthesis and analysis using STATA version 13.1 (StataCorp LLC, College Station, TX, USA). The hierarchical summary receiver operating characteristic (hsROC) curve was constructed using sensitivity, specificity, and the parameters of the bivariate normal distribution in order to assess the diagnostic performance of uKIM-1 and serum/plasma YKL-40 in the early diagnosis of DN. To achieve this, the absolute numbers of TPs, FPs, FNs, and TNs had to be extracted from the data in order to construct the 2 × 2 table. If not provided in the studies, these parameters were estimated from raw data such as the means of uKIM-1 and YKL-40 and their SDs, assuming a normal distribution. When such data were not given, we calculated the counts of the 2 × 2 table equivalent information given by sensitivity, specificity, PPV, NPV, or the AUC.

The model of the bivariate meta-analysis was based on the original bivariate approach by van Houwelingen [18], as it was later modified for the synthesis of diagnostic data [19,20]. The hsROC curve was constructed according to the method proposed by Harbord et al. [21], which uses logit-transforms of true positive rate (TRR and false positive rate (FPR) and is based on simple linear regression of their differences (D), which is the diagnostic log odds ratio (logDOR) on their sum (S), which is a function of the test threshold.

If the 95% confidence interval (CI) was reported, *SE* was estimated according to the recommendations of the Cochrane Handbook [22] using the formula:

(1) SE=(upperlimit−lowerlimit)/3.92

If the outcome measures were reported as median (*M*) and inter-quartile range (*IQR*), mean and *SD* values were estimated according to the recommendations of the Cochrane Handbook [22]. In that case, the median was used as an estimator of the mean, whereas the *SD* was calculated using:

(2) $$SD=\frac{IQR}{1.35}$$

In case the outcome measures were reported as median (*M*) and range, mean and *SD* values were estimated using the methods and the guidelines described by Hozo and co-workers [23]. For small sample sizes (*n* < 25), we used the formula:

(3) $$\bar{x}=\frac{\min+2M+max}{4}$$

For *n* > 25, the median itself was used, as it is considered to be better estimator of the mean. Concerning the *SD*, for small sample sizes (*n* < 15), we used the formula:

(4) $$SD^{2}=\frac{1}{12}\left(\frac{{\left(min+2M+max\right)}^{2}}{4}+{\left(max-min\right)}^{2}\right)$$

whereas, for larger ones, the formula was:

(5) $$SD=\frac{R}{4}$$

Due to their skewed distribution, the mean values of uKIM-1 and YKL-40 were log transformed according to the guidelines described by Higgins and co-workers [24]. Specifically, to convert ${\bar{x}}_{i}$ and $s_{x,i}$ to an approximate mean and *SD* on the log-transformed scale, we used the following formulas:

(6) $${\overset{´}{z}}_{i}=ln\left({\overset{´}{x}}_{i}\right)-1/2ln\left(\frac{s_{x,i}{}^{2}}{{\overset{´}{{\overset{´}{x}}_{i}}}^{2}}+1\right)\left(i=1,2\right)$$

and

(7) $$s\prime_{z,i}=\sqrt{ln\left(\frac{s^{2}{}_{x,i}}{{\overset{´}{x}}_{i}{}^{2}}+1\right)}\left(i=1,2\right)$$

where ${\bar{x}}_{i}$ and $s_{x,i}$ are the mean and *SD* of our measurements of interest.

By varying the decision thresholds (log-cutoff values), the normal distribution probability was calculated for each status variable estimating thus the TP, FN, FP, and TN values. The optimal cut-off was estimated by calculating the Youden’s index [25] and estimating its maximum value as follows:

Y = Sensitivity + Specificity − 1(8)

The interpretation of the result of the area under curve was also calculated and was done according to the guidelines suggested by Swets [26] as follows: low (0.5 ≥ AUC ≤ 0.7), moderate (0.7 ≥ AUC ≤ 0.9), and high (0.9 ≥ AUC ≤ 1.0) accuracy.

The inter-study heterogeneity was appraised using Cohran’s Q and I^2^ statistics [27]. The I^2^ statistic reflects the levels of heterogeneity as follows: 25%, 50%, and 75% indicating low, moderate and high heterogeneity, respectively. To address possible heterogeneity, a sensitivity analysis was performed. Deek’s funnel plot was used to evaluate publication bias by using linear regression of logodds ratios on the inverse root of the effective sample sizes [28].

Finally, correlation analysis was performed to assess the association of uKIM-1 and YKL-40 with eGFR, HbA1c, and uACR for each study independently. Subsequently, univariate random effect meta-analysis of the resulted individual correlation (r) coefficients was performed in order to yield the overall r coefficient.

## 3. Results

### 3.1. Electronic Search Results and Study Characteristics

**Figure 1:**
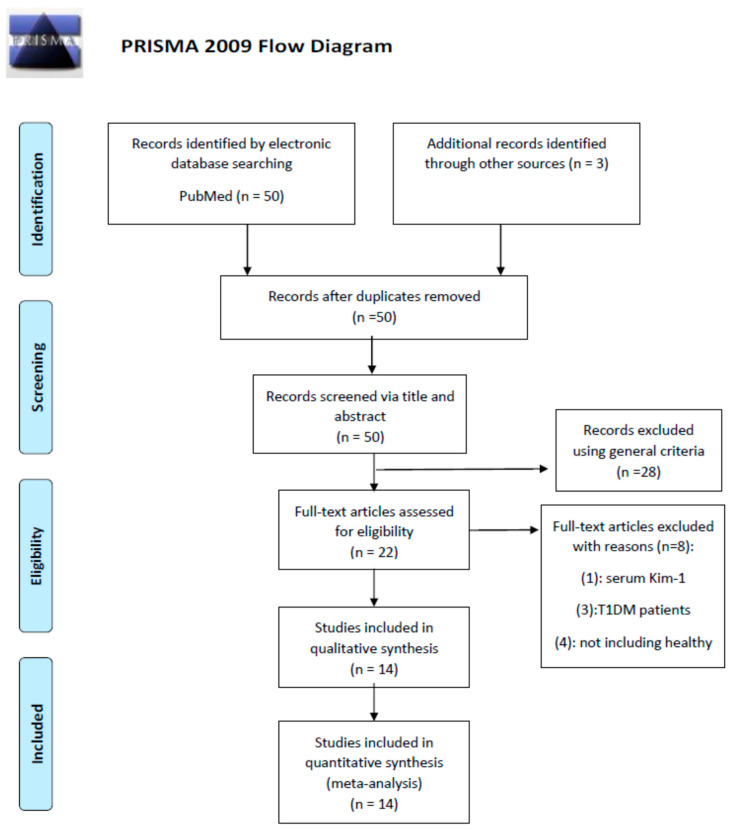
The PRISMA flow diagram for the included studies.

A total of 53 titles and abstracts were initially reviewed in the electronic databases as well as in other sources after the primary selection. A total of 39 articles were excluded from the meta-analysis; three for duplication, 28 as they did not meet the inclusion criteria or had inappropriate data, seven because the target group was not appropriate (four due to lack of control/healthy group and three with T1DM patients), and one because it included the measurements for serum KIM-1. Fourteen articles ultimately satisfied the inclusion criteria of which eight had data for uKIM-1 and six for the YKL-40 biomarker. A total of 196 controls and 282 T2DM patients with normoalbuminuria, with a weighted average age of 49.6 and 55.1, respectively, were enrolled in these studies involving the uKIM-1 biomarker. Studies involving the YKL-40 biomarker included 402 controls and 483 T2DM patients with normoalbuminuria, with a weighted average age of 53.6 and 54.6, respectively. A summary of the selection of studies is illustrated by the PRISMA flow diagram (Figure 1). Table 1 and Table 2 represent the detailed information of each study included in the meta-analysis for the two biomarkers uKIM-1 and YKL-40, respectively. Supplementary Tables S1 and S2 show the descriptive statistical results of the controls and T2DM patients with normo-, micro-, and macroalbuminuria in studies involving uKIM-1 and YKL-40, respectively.

### 3.2. Quality Assessment of the Included Studies

The methodological quality of the studies according to QUADAS is summarized in Supplementary Tables S3 and S4. Overall, the applicability concern in the four categories regarding the included studies for both biomarkers was low. Specifically, in the studies concerning uKIM-1, the QUADAS results showed a possible bias in the patient selection. Specifically, one study enrolled only female patients and another did not mention the inclusion/exclusion criteria for T2DM patients as far as other co-existing diseases were concerned. The bias regarding flow and timing was introduced due to the different way of storing the urine samples in a few studies. Long-term storage (−80 °C or −70 °C) of the urine samples may have influenced uKIM-1 analysis. For studies involving the YKL-40 biomarker, the unclear risk of bias in patient selection was introduced in one study, as it did not mention the inclusion/exclusion criteria of the diabetic patients. Regarding flow and timing, bias was introduced again due to the fact that four studies used long-term storage of the serum sample until it was assayed. The concern regarding applicability of the reference standard in one study was due to the fact that the threshold used to categorize the patients was slightly different from that reported in the rest of the studies.

### 3.3. Data Synthesis: Contingency Table, Diagnostic Performance, Hierarchical Summary Receiver Operating Characteristic Curve, Sensitivity Analysis, Publication Bias and Correlation Analysis

Table 3 lists the TP, FN, FP, and TN values, the paired sensitivity and specificity along with the corresponding 95% confidence interval (CI) for the diagnosis of early DN in T2DM patients for each individual study for both biomarkers. The summary results of the sensitivity analysis for YKL-40 are also listed in Table 3. Table 4 summarizes in detail the diagnostic and prognostic estimates including pooled sensitivity, specificity, positive and negative likelihood ratio, diagnostic odds ratio area under curve along with their 95%CI as well as the I^2^ for heterogeneity and the *p* value of publication bias for uKIM-1 and YKL-40.

**Figure 2:**
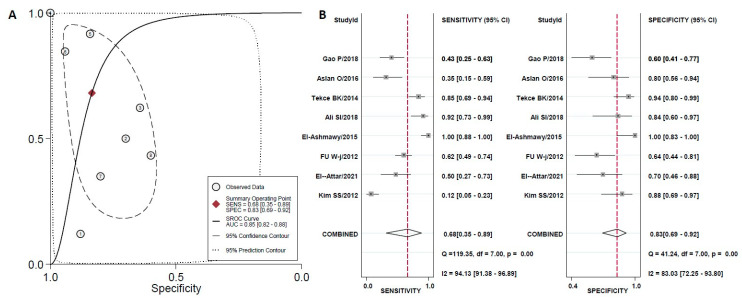
(**A**) The hierarchical Summary Receiver Operating Characteristic curve curve of uKIM-1to discriminate controls (healthy individuals) from normoalbuminuric T2DM patients. The straight line represents the hSROC curve; the circle represents each of the analyzed studies; the diamond shape represents the point estimate to which overall sensitivity and specificity correspond. (**B**) Forest plot for sensitivity and specificity.

**Figure 3:**
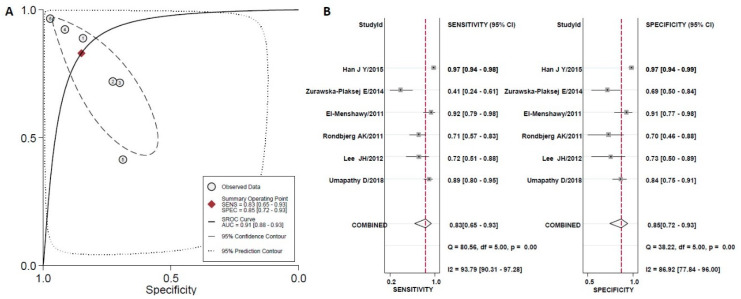
(**A**) The hierarchical Summary Receiver Operating Characteristic curve of YKL-40 to discriminate controls (healthy individuals) from normoalbuminuric T2DM patients. The straight line represents the hSROC curve; the circle represents each of the analyzed studies; the diamond shape represents the point estimate to which overall sensitivity and specificity correspond. (**B**) Forest plot for sensitivity and specificity.

The estimated overall sensitivity of urinary uKIM-1 for the diagnosis of early DN was 0.68 (95%CI 0.35–0.89) and specificity was 0.83 (95% CI 0.69–0.92) with a DOR of 11 (95%CI 2–75), as shown in Figure 2B. For YKL-40, the corresponding values were 0.83 (95%CI 0.65–0.93) and 0.85 (95%CI 0.72–0.93), respectively, with DOR of 28 (95%CI 5–156) (Figure 3B). There was strong heterogeneity between studies in both sensitivity and specificity for both biomarkers, as indicated by I^2^ indexes of 94.1% and 83.0% for uKIM-1 and of 94.0% and 83.0% for YKL-40, respectively.

A hSROC curve was obtained for each biomarker and the AUC was estimated along with the 95% CI. The hSROC results showed that the AUC of urinary uKIM-1 was 0.87 (0.83–0.89), suggesting a moderate diagnostic accuracy of uKIM-1 for DN diagnosis (Figure 2A). The AUC for YKL-40 was 0.91 (0.88–0.93), suggesting that the accuracy of YKL-40 for DN diagnosis is high (Figure 3A).

We performed sensitivity analysis by excluding the study of Rondbjerg et al. from the meta-analysis concerning the YKL-40 biomarker, as the categorization of DN patients was slightly different from the ones described in the rest of the studies and may have potentially biased the categorization of the T2DM patients with DN. The results of the sensitivity analysis yielded an AUC of 0.92 (0.90–0.94), as shown in Table 4.

The evaluation of publication bias according to Deek’s funnel plot asymmetry test showed potential bias only in the studies measuring YKL-40 in T2DM patients (*p* < 0.05). However, the publication bias yielded a *p*-value of 0.05 when the study of Rondbjerg et al. was excluded (Table 3). Statistically significant publication bias was not found in studies with the uKIM-1 data (*p* = 0.72).

Finally, Supplementary Table S5 summarizes the results of the correlation analysis of uKIM-1 and YKl-40 with various clinical parameters including the indicators of renal damage (eGFR and uACR). Overall, a statistical positive association was found between uKIM-1 and YKL-40 with uACR. An inverse significant correlation was found between uKIM-1 and eGFR. This correlation was not present in the YKL-40 biomarker. No association was found between uKIM-1 and YKL-40 with the duration of diabetes and HbA1c. YKL-40 and eGFR correlated in a negative, but not statistically significant manner.

## 4. Discussion

DN is the leading cause of end-stage renal disease, affecting the morbidity and mortality in patients with diabetes. Nowadays, microalbuminuria assessed by measuring uACR, a common standard to test DN, displays numerous limitations that affect the early diagnosis and prognosis of DN [42]. Specifically, microalbuminuria is diagnosed once significant glomerular damage has occurred and does not necessarily lead to renal deterioration as it has been demonstrated that some diabetic patients with microalbuminuria shift back to normoalbuminuria with simultaneous reduction in urinary albumin excretion rate [43]. In addition, it is shown that even in diabetic patients with normal urinary excretion, severe damage is still present and nephropathy sometimes occurs in normoalbuminuric patients. Moreover, recent studies have shown that tubulointerstitial and glomerular injuries as well as inflammation play an important role in the pathogenesis of DN. In order to enhance the ability to predict the occurrence of DN and provide an earlier clinical approach to its treatment, more and more effort by researchers has been made to discover novel biomarkers prior to uACR. In a previous meta-analysis, we examined the performance characteristics of NGAL both in serum and in urine and concluded that it could be used as a valuable biomarker for the early detection of DN in type 1 and type 2 diabetic patients [6]. In the present meta-analysis, we synthesized all the published studies that examined the performance characteristics of one tubular biomarker, the urinary biomarker KIM-1 and one inflammatory marker, the serum/plasma YKL-40 biomarker in order to evaluate their performance in the prediction of early DN in T2DM patients.

Of all the identified studies of diabetic patients at risk for DN, 14 could be meta-analyzed for the diagnosis of DN concerning both biomarkers. Overall, our results showed that uKIM-1 is a moderate biomarker for predicting early DN in patients with type 2 diabetes, according to the value of AUC. On the other hand, YKL-40 seems to perform better in the diagnosis of DN in T2DM patients.

To be more specific, meta-analysis of ROC curves revealed that tubular biomarker uKIM-1can be useful to assess early diabetic renal damage. This finding supports the hypothesis that tubular involvement precedes glomerular involvement in diabetic kidney disease. Accordingly, uKIM-1 seems to be increasing in the very early stage of diabetic nephropathy even before the appearance of pathological albuminuria, suggesting that tubules are damaged at the initial stage of diabetes. This assumption is enhanced by the results of the correlation analysis that showed that uKIM-1 had a statistically significant positive correlation with uACR, the measurable sign of renal diabetic impairment, a result which coincides with other published studies [34,35]. uKIM-1 also had a significant negative correlation with eGFR, the indicator of deterioration of renal function, a result which is in harmony with a previously reported result [32]. However, we did not find any correlation of the biomarker with the duration of diabetes.

In addition, serum/plasma YKL-40 levels seem to increase in parallel with the development of DN and seem to be elevated in T2DM patients when compared with the control groups even before albuminuria appears. This finding is in accordance with other studies that associate chronic low-grade inflammation with the occurrence and progression of albuminuria [44]. Not surprisingly, a significant correlation was found between YKL-40 and uACR. However, no profound correlation was found between the marker and the decline of eGFR, a finding that has been also reported in a previous study [38]. YKL-40 did not correlate as being statistically significant with the duration of diabetes.

However, for both markers, the meta-analysis had a high inconsistency index denoting a high degree of heterogeneity. The heterogeneity may be due to the study design, the population setting, the sample and methodology used to estimate the biomarkers, and the time point of measurement of the biomarkers. In more detail, all studies had a uniform definition of microalbuminuria using as reference standard the urinary albumin-creatinine excretion, but different methods for detecting the concentration of creatinine and different ways of urine collection, either 24-h urine or random spot urine (and not first-morning void), which might have influenced the uACR measurement as well as the uKIM-1 measurement. In addition, in some studies, measurement of uKIM-1 was achieved after long-term storage at −80 °C of the urine samples and not immediately on the same day of the sample collection. This parameter, in conjunction with the pre-mentioned factor for the reference standard (uACR) may have also introduced potential bias as also assessed by the quality study (QUADAS). The same can apply for YKL-40 where some studies used plasma and others serum YKL-40 as an index test. In particular, to explore the heterogeneity present in the YKL-40 data, we used sensitivity analysis by excluding a study with a slightly different definition of DN (reference standard) and repeated the meta-analysis. The diagnostic significance of YKL-40 was not expressively altered.

In addition, the chief methodology of the biomarker’s measurements was based on enzyme-linked immunosorbent assay (ELISA). However, a series of independent immunoassays have been used with variable antibodies and reagents, leading to differences in the measurements of the biomarkers.

Moreover, a certain degree of publication bias was found in the analysis concerning YKL-40 for DN diagnosis, which was however eliminated in the sensitivity analysis. No publication bias was found in the analysis concerning uKIM-1 for predicting DN.

In conclusion, our study provides additional data about the role of two biomarkers, uKIM-1 and YKL-40, in the development of early diabetic nephropathy, with both being promising biomarkers in the diagnosis of the disease as they can be detectable in early stages and subclinical diseases. However, whether they can be used as new markers for diagnosing and monitoring DN independent of albuminuria, needs further investigation. Prospective and multicenter studies are needed to confirm whether uKIM-1 and YKL-40 can act as prognostic markers toward the progression of albuminuria in T2DM patients with increased levels, independent of their albuminuria status.

## Figures and Tables

**Table 1:** Detailed characteristics of the included studies in the meta-analysis, concerning the uKIM-1 biomarker for controls and T2DM patients with normoalbuminuria.

First Author’ s Name, Year (Reference)	Country	Sample Size (n)		Sex (%Male)		Age		Definition of Albuminuria for T2DM Patients	Method of uKIM-1 Measurement	Variables Provided
		Controls	Patients	Controls	Patients	Controls	Patients			
Kim SS, 2012 [29]	Republic of Korea	25	58	56.0	39.6	50.9	57	Normoalbuminuria: uACR < 30 mg/g	ELISA (R &D systems)	Median + IQR
El-Attar HA, 2017 [30]	Egypt	20	20	45.1	50.0	39.5	39.5	Normoalbuminuria uACR < 30 mg/g	ELISA	Median+ min, max
Fu Wen-jin, 2012 [31]	China	28	61	46.4	-	48.3	-	Normoalbuminuria uACR < 30 mg/g	ELISA (Quantikine R & D)	Median + IQR
El-Ashmawy NE., 2015 [32]	Egypt	20	30	50.0	33.3	51.6	60.2	Normoalbuminuria uACR < 30 mg/g	ELISA (Adibo Bioscience)	Mean + SD
Ali SI., 2017 [33]	Egypt	19	24	42.8	-	45.0	-	Normoalbuminuria uACR < 30 mg/g	ELISA (Glory Diagnostics)	Mean + SD
Kin Tekce B, 2014 [34]	Turkey	34	39	47.0	46.1	59	62	Normoalbuminuria uACR < 30 mg/g	ELISA (Aviscera Bioscience)	Mean + SD
Aslan O, 2014 [35]	Turkey	20	20	0	0	48.4	52.1	Normoalbuminuria uACR < 30 mg/g	ELISA (USCN)	Mean + SD
Gao P, 2018 [36]	USA	30	30	50.0	53.3	48.1	50.1	Normoalbuminuria uACR < 30 mg/g	ELISA (USCN)	Median + IQR

**Table 2:** Detailed characteristics of the included studies in the meta-analysis, concerning the YKL-40 biomarker for controls and T2DM patients with normoalbuminuria.

First Author’ s Name, Year (Reference)	Country	Sample Size (n)		Sex (%Male)		Age		Definition of Albuminuria for T2DM Patients	YKL-40	Method of uKIM-1 Measurement	Variables Provided
		Controls	Patients	Controls	Patients	Controls	Patients				
Umapathy D, 2018 [37]	India	83	81	52.6	60.5	54.1	54.1	Normoalbuminuria: uACR < 30 mg/g	Plasma	Immunoassay (Bio-PlexPro^™^)	Median + range
El-Menshawy N, 2011 [11]	Egypt	35	39	54.3	48.7	49.3	52.5	Normoalbuminuria uACR < 30 mg/g	Serum	EIA (METRA, QUIDEL)	Mean + SD
Rondbjerg AK, 2011 [38]	Denmark	20	49	60.4	44.8	57.1	61.3	Normoalbuminuria uACR < 22.12 mg/g	Serum	ELISA (Quidel, USA)	Median + IQR
Zurawska-Plaksej E, 2014 [39]	Poland	32	29	37.5	37.9	61.0	62.9	Normoalbuminuria uACR < 30 mg/g	Plasma	ELISA (MicroVue, Quidel)	Mean + SD
Han JY, 2015 [40]	China	210	260	48.4	50.7	53.4	52.8	Normoalbuminuria uACR < 30 mg/g	Serum	ELISA (Bio-Technology)	Median + IQR
Lee JH, 2012 [41]	South Korea	22	25	59.1	44	52.4	55.6	Normoalbuminuria uACR < 30 mg/g	Plasma	ELISA (R&D Systems)	Median + IQR

**Table 3:** Contingency table for uKIM-1 and YKL-40 inT2DM patients along with paired sensitivity and specificity of individual studies for the diagnosis of early DN in each study included in the meta-analysis.

Study	True Positive	False Negative	True Negative	False Positive	Sensitivity (95%CI)	Specificity (95%CI)
uKIM-1: control vs. normoalbuminuric T2DM patients						
Kim SS, 2012	7	51	22	3	0.12 (0.05–0.23)	0.88 (0.69–0.97)
El-Attar HA, 2017	10	10	14	6	0.50 (0.27–0.73)	0.70 (0.46–0.88)
Fu Wen-jin, 2012	38	23	18	10	0.62 (0.49–0.74)	0.64 (0.44–0.81)
El-Ashmawy NE., 2015	30	0	20	0	1.00 (0.88–1.00)	1.00 (0.83–1.00)
Ali SI., 2017	22	2	16	3	0.92 (0.73–0.99)	0.84 (0.60–0.97)
Kin Tekce B, 2014	33	6	32	2	0.85 (0.69–0.94)	0.94 (0.88–0.99)
Aslan O, 2014	7	13	16	4	0.35 (0.15–0.59)	0.80 (0.56–0.94)
Gao P, 2018	13	17	18	12	0.43 (0.25–0.63)	0.60 (0.41–0.77)
YKL-40: control vs. normoalbuminuric T2DM patients						
Umapathy D, 2018	72	9	70	13	0.89 (0.80–0.95)	0.84 (0.75–0.91)
El-Menshawy N, 2011	36	3	32	3	0.92 (0.79–0.98)	0.91 (0.77–0.98)
Rondbjerg AK, 2011	35	14	14	6	0.71 (0.57–0.83)	0.70 (0.46–0.88)
Zurawska-Plaksej E, 2014	12	17	22	10	0.41 (0.24–0.61)	0.69 (0.77–0.98)
Han JY, 2015	251	9	204	6	0.97 (0.94–0.98)	0.97 (0.94–0.99)
Lee JH, 2012	18	7	16	6	0.72 (0.51–0.88)	0.73 (0.50–0.89)

**Table 4:** Pooled diagnostic and prognostic accuracy of uKIM-1 and YKL-40 for the diagnosis of early DN in T2DM patients. Results of sensitivity analysis, *p* value of publication bias, and I^2^ for heterogeneity are also given.

No of Studies	Sensitivity (95% CI)	I^2^(%)	Specificity (95%CI)	I^2^ (%)	PLR (95%CI)	NLR (95% CI)	DOR (95% CI)	AUC (95%CI)	*p* Value (Publication Bias)
uKIM-1: controls vs. T2DM patients with normoalbuminuria									
9	0.68 (0.35–0.89)	94.1	0.83 (0.69–0.92)	83.0	4.1 (1.5–11.0)	0.38 (0.14–1.06)	11 (2, 75)	0.85 (0.82–0.88)	0.74
YKL-40: controls vs. T2DM patients with normoalbuminuria									
6	0.83 (0.65–0.93)	94.0	0.85 (0.72–0.93)	87.0	5.5 (2.4–12.8)	0.20 (0.08–0.55)	28 (5, 156)	0.91 (0.88–0.93)	0.01
YKL-40: controls vs. T2DM patients with normoalbuminuria-Sensitivity analysis									
5	0.85 (0.64–0.95)	94.8	0.87 (0.73–0.94)	88.1	6.5 (2.5–16.5)	0.18 (0.06–0.52)	37 (5, 269)	0.92 (0.90–0.94)	0.05

Abbreviations: PLR: positive likelihood ratio, NLR: negative likelihood ratio, DOR: diagnostic odds ratio, AUC: area under the curve.

## Supplementary Materials

The following are available online at https://www.mdpi.com/2075-4418/10/11/909/s1, Table S1: Descriptive statistical results of thee controls and T2DM patients with normo-, micro- and macroalbuminuria in studies involving KIM-1, Table S2: Descriptive statistical results of controls and T2DM patients with normo-, micro- and macroalbuminuria in studies involving YKL-40, Table S3: Risk of bias and applicability concern graph for the studies concerning uKIM-1. Each risk of bias item of all included studies is represented as percentage (%), Table S4: Risk of bias and applicability concern graph for the studies concerning YKL-40. Each risk of bias item of all included studies is represented as percentage (%), Table S5: Correlation analysis of KIM-1 and YKL-40 with eGFR, HbA1c, and UACR.
