# Supplementary material for: Diagnostic Performance of Biomarkers Urinary KIM-1 and YKL-40 for Early Diabetic Nephropathy, in Patients with Type 2 Diabetes: A Systematic Review and Meta-Analysis
Source: Diagnostics (Basel). 2020 Nov 7;10(11):909. doi: 10.3390/diagnostics10110909 (PMC7695026; doi:10.3390/diagnostics10110909)
Supplement: Supplementary file 1 [file diagnostics-10-00909-s001.pdf]

**Table S1.** Descriptive statistical results of controls and T2DM patients with normo-, micro- and macroalbuminuria, in studies involving KIM-1.

| Type of participants | Controls (healthy) | Type 2 Diabetes with normoalbuminuria | Type 2 Diabetes with microoalbuminuria | Type 2 Diabetes with macroalbuminuria |
|---|---|---|---|---|
| **no. of participants** | 196 | 282 | 92 | 135 |
| **Sex (%male)** | 48.4 | 43.5 | 47.2 | 52.1 |
| **Age** | 49.6 | 55.1 | 56.8 | 59.2 |
| **eGFR(mL/min/1,73m$^2$)** | 105.9 | 99.0 | 96.6 | 73.3 |
| **HbA1c (%)** | 4.5 | 7.6 | 8.0 | 8.7 |
| **UACR (mg/g Cr)** | 7.9 | 12.4 | 82.7 | 7521.0 |

eGFR: estimated glomerular filtration rate, HbA1c: glycated hemoglobin A1c, UACR: urinary albumin-creatinine ratio.

**Table S2.** Descriptive statistical results of controls and T2DM patients with normo-, micro- and macroalbuminuria, in studies involving YKL-40.

| Type of participants | Controls (healthy) | Type 2 Diabetes with normoalbuminuria | Type 2 Diabetes with microoalbuminuria | Type 2 Diabetes with macroalbuminuria |
|---|---|---|---|---|
| **no. of participants** | 402 | 483 | 457 | 416 |
| **Sex (%male)** | 50.1 | 50.5 | 51.3 | 52.5 |
| **Age** | 53.6 | 54.6 | 55.1 | 56.0 |
| **eGFR(mL/min/1,73m$^2$)** | 105.3 | 86.8 | 77.8 | 67.1 |
| **HbA1c (%)** | 5.4 | 8.3 | 8.6 | 8.8 |
| **UACR (mg/g Cr)** | 8.8 | 12.4 | 81.7 | 740.5 |

eGFR: estimated glomerular filtration rate, HbA1c: glycated hemoglobin A1c, UACR: urinary albumin-creatinine ratio.

**Table S3.** Risk of bias and applicability concern graph for the studies concerning uKIM-1. Each risk of bias item of all included studies, is represented as percentage (%).

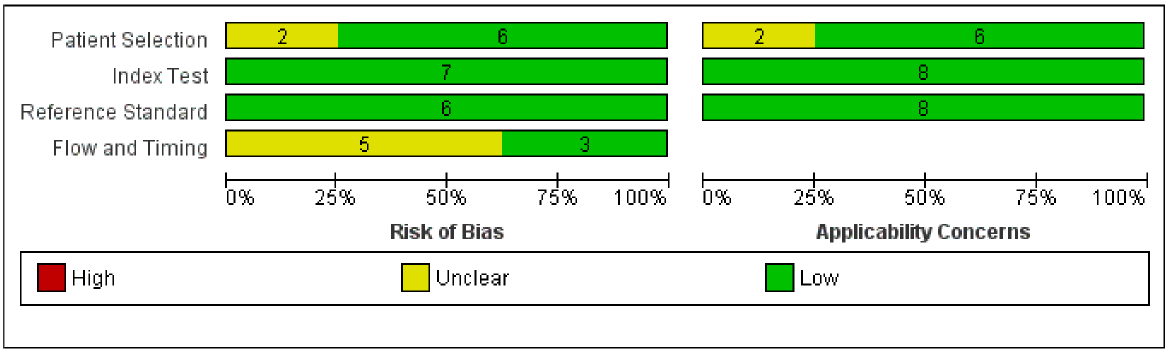


**Table S4.** Risk of bias and applicability concern graph for the studies concerning YKL-40. Each risk of bias item of all included studies, is represented as percentage (%).

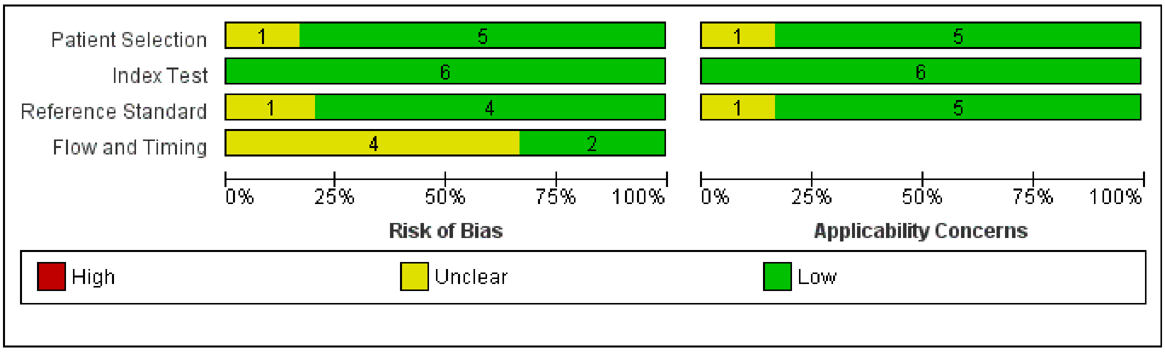


`

**Table S5.** Correlation analysis of KIM-1 and YKL-40 with eGFR, HbA1c and UACR.

| **Parameters** | **n. of studies** | **KIM-1** | | **n. of studies** | **YKL-40** | |
|---|---|---|---|---|---|---|
| | | **r** | **p value** | | **r** | **p value** |
| duration of diabetes | 5 | 0.56 | 0.161 | 5 | 0.75 | 0.066 |
| eGFR (mL/min/1,73m$^2$) | 4 | -0.95 | 0.001 | 5 | -0.68 | 0.148 |
| HbA1c (%) | 4 | 0.83 | $<10^{-4}$ | 6 | 0.86 | $<10^{-4}$ |
| UACR (mg/g Cr) | 5 | 0.91 | 0.003 | 4 | 0.88 | 0.004 |

eGFR: estimated glomerular filtration rate, HbA1c: glycated hemoglobin A1c, UACR: urinary albumin-creatinine ratio. p value <0.05 denotes a statistically significant result.
